# Gustatory dysfunction under talquetamab therapy in multiple myeloma: a cross-sectional study of patient-reported course, nutritional changes, and quality of life

**DOI:** 10.1007/s00520-026-11288-4

**Published:** 2026-10-05

**Authors:** Anna Fleischer, Carolin Burger, Magdalena Roll, Franziska Panther, Patrick-Pascal Strunz, Jessica Peter, Sofie-Katrin Kadel, Anja Gesierich, Julia Mersi, Johannes Waldschmidt, Martin Kortüm, Hermann Einsele, Imad Maatouk, Leo Rasche

**Affiliations:** Department of Internal Medicine II, University Hospital Würzburg, Oberdürrbacherstr. 6, 97080 Würzburg, Germany

**Keywords:** Talquetamab, Multiple myeloma, Dysgeusia, Appetite, Quality of life, Supportive care

## Abstract

The purpose of this study is to characterize patient-reported taste disturbances, recalled symptom course, nutritional changes, and quality of life in patients with relapsed/refractory multiple myeloma treated with talquetamab. This single-center cross-sectional study combined taste-strip testing with a study-specific questionnaire, EORTC QLQ-C30, and PHQ-4 in 32 patients. Analyses used recorded aggregate correct-identification counts without a normative hypogeusia classification. Symptom timing and changes since treatment initiation were recalled at one assessment. All patients reported taste disturbances during therapy. The median identification count was 2.5 (interquartile range 0.75–7.25); eight patients had no correct identifications. Twenty-three (71.9%) described onset within 14 days. Current appetite reduction was reported by 20 patients (62.5%), whose mean identification count was lower than that of the other 12 patients (2.45 versus 7.83; mean difference − 5.38, 95% confidence interval − 8.20 to − 2.57; *p* = 0.0006). Sixteen patients (50.0%) reported directional weight loss, with a median loss of 4.5 kg. Ten (31.3%) had considered stopping talquetamab; seven explicitly cited taste disturbance. Mean global health status was 50.5/100 and correlated with identification count (*r* = 0.406; *p* = 0.021). Taste disturbances co-occurred with substantial nutritional concerns. Lower taste-identification counts were associated with current appetite reduction and lower global health status. The findings support attention to taste-related burden but do not establish causality, sustained recovery, or effects on adherence. Prospective evaluations of supportive care are needed.

**Supplementary Information:**

The online version contains supplementary material available at https://doi.org/10.1007/s00520-026-11288-4.

## Introduction

Talquetamab (TAL), a GPRC5D × CD3 bispecific antibody, has demonstrated antimyeloma activity in heavily pretreated relapsed/refractory multiple myeloma (MM), while taste disturbances form part of its characteristic adverse-event profile [1]. Clinical reports have described reduced gustatory performance, xerostomia, and weight loss during treatment [2, 3]. These symptoms warrant attention alongside disease control [4] because the experience of eating is relevant to both nutritional intake and daily well-being.

Taste-related burden is not fully described by a binary adverse-event label. Patients may perceive food differently, lose enjoyment of meals, modify food choices [5], or reconsider treatment despite an antimyeloma response. Chemotherapy research illustrates the heterogeneity of chemosensory changes and their relationship with food perception [6]. Earlier comparative work from our group examined taste disturbances across TAL, BCMA-targeted bispecific antibodies, and high-dose melphalan [7]. The present report focuses instead on within-cohort relationships between recorded taste performance, appetite, and patient-reported burden in TAL-exposed patients; no comparative estimates from the earlier report were pooled with these data.

The objective was to describe taste-identification performance, patient-reported onset and course of taste disturbances, appetite and weight changes, food preferences, quality of life (QoL), and thoughts about stopping treatment, and to explore their associations. Temporal information was obtained retrospectively at a single assessment, rather than by observing individual trajectories prospectively.

## Methods

### Study design and participants

This single-center cross-sectional observational study was conducted at University Hospital Würzburg, Germany, with assessments documented in 2024–2025. Eligible adults had relapsed/refractory MM, had received at least one TAL dose after the step-up phase, and could provide informed consent and participate in the assessments. Patients receiving TAL and patients previously treated with TAL were eligible. Exclusion criteria included conditions preventing reliable participation, active oral infection or severe dental disease at testing, and allergy to the test substances. The analysis included all 32 available paired taste-testing and questionnaire records. Demographic and treatment information was taken from the study records; explicitly different staging systems were not combined.

### Assessment procedure and taste testing

Assessments took place during routine inpatient or outpatient visits. Patients were instructed to avoid food, drinks other than water, smoking, and chewing gum for at least one hour before testing. Taste strips (Burghart Messtechnik GmbH) were used to assess taste identification [8–10]. The administered panel included sweet, sour, salty, bitter, and umami stimuli at four concentrations, together with neutral control strips. Strips were applied to the tongue, the mouth was rinsed with water between presentations, and the perceived taste or absence of taste was recorded. Questionnaires followed the taste assessment, with assistance as needed.

The analytic records retained an aggregate number of correct identifications, but not the underlying responses by quality and concentration. The recorded count was therefore analyzed as a continuous performance measure. A standard four-quality 0–16 total could not be verified from these records; norm-referenced hypogeusia categories and quality-specific identification rates were not reconstructed. A recorded zero was reported descriptively, not diagnosed as ageusia.

### Patient-reported measures

The study-specific questionnaire covered symptoms experienced since TAL initiation, recalled onset and improvement, current taste-related impairment, appetite, weight change, food preferences, coping strategies, and consideration of stopping TAL. It was not a validated dysgeusia or adherence instrument. Current impairment was elicited by an open question. Explicit intensity descriptions were coded from 0 (none) to 4 (very severe), with nonclassifiable or missing responses left ungraded. Food categories were nonexclusive descriptive codes. Coding rules and source-item distinctions are detailed in Online Resource 1.

Appetite reduction was contrasted with no current reduction, including one report of initial reduction followed by normalization. Directional weight loss was distinguished from gain, stable weight, and nondirectional fluctuation. A reported 3–4-kg loss was represented by its midpoint for descriptive summaries. Questions about actual consideration of stopping and its stated reasons were kept separate from hypothetical reasons for stopping. Discordant improvement items were described separately rather than combined into a recovery endpoint. Inconsistent documentation of current treatment status precluded analyses stratified by ongoing versus discontinued TAL.

### Quality of life and distress

EORTC QLQ-C30 version 3.0 scores were calculated from individual questionnaire items [11, 12]. The workbook explicitly coded items 1–28 from 0 to 3; these were aligned with the standard scoring algorithm. Items 29–30 retained their 1–7 coding. Scores range from 0 to 100, with higher values representing better global health/functioning or greater symptom burden. One pain-item value outside the permitted range was treated as missing; the pain score used the remaining valid item under the EORTC half-item rule. PHQ-4 items were summed to a 0–12 score and classified as none/minimal (0–2), mild (3–5), moderate (6–8), or severe (9–12) distress [13].

### Statistical analysis

Continuous data are summarized as mean (sample SD) or median (IQR; linearly interpolated 25th–75th percentiles), and categorical data as counts and percentages with explicit denominators. Welch’s *t*-tests compared continuous outcomes between groups. Pearson correlations, including the point-biserial correlation for appetite status, assessed continuous associations; Spearman correlations were used for the ordinal impairment grade. Binary comparisons used two-sided Fisher exact tests. Mean differences and Pearson correlations are accompanied by 95% confidence intervals where reported. Missing, invalid, and nonclassifiable entries were excluded only from the relevant analysis; absent narrative information was not assigned a numerical clinical outcome.

All analyses were exploratory and unadjusted; *p* values are nominal, without multiplicity correction. No multivariable or causal model was fitted. Sensitivity analyses used the Mann–Whitney *U* test for taste counts by appetite status, Spearman correlation for taste counts and global health, and alternative handling of the resolved appetite report, ambiguous weight/burden entries, and the incomplete pain scale.

## Results

### Participant and treatment characteristics

The 32 participants had a median age of 66.5 years (IQR 57.5–72.0; range 36–75); 21 (65.6%) were male. Twenty-three records contained an interpretable ISS stage (I, *n* = 12; II, *n* = 5; III, *n* = 6). Two entries explicitly used R-ISS, five used Durie–Salmon staging, and two were not classifiable. Concomitant antimyeloma agents were explicitly documented in five records. Polyneuropathy was recorded in 19 participants (59.4%), COVID-19 history in eight (25.0%), and reflux in six (18.8%) (Table 1).

**Table 1:** Participant characteristics and recorded treatment context

Characteristic	Value
Participants	32
Age, years: median (IQR); range	66.5 (57.5–72.0); 36–75
Male/female	21 (65.6%)/11 (34.4%)
Stable partnership or married	26 (81.3%)
Single/divorced/widowed	2/1/3
Education	
University degree	16 (50.0%)
Upper secondary qualification	1 (3.1%)
Intermediate secondary qualification	7 (21.9%)
Lower secondary/primary qualification	8 (25.0%)
Staging entries	
ISS I/II/III (23 classifiable records)	12/5/6
Explicitly labeled R-ISS (II/III)	1/1
Explicitly labeled Durie–Salmon IIIA	5
No classifiable staging entry	2
Recorded treatment-cycle number (*n* = 29)	Median 2 (IQR 1–4); range 1–18
No interpretable cycle number	3 (9.4%)
Documented concomitant antimyeloma agents	5 (15.6%)
Recorded comorbidities	
Polyneuropathy	19 (59.4%)
COVID-19 history	8 (25.0%)
Gastroesophageal reflux disease	6 (18.8%)
Diabetes mellitus	2 (6.3%)
Stroke	3 (9.4%)
Comorbidity item not completed	3 (9.4%)

Percentages use the full cohort (*n* = 32). *IQR*, interquartile range; *ISS*, International Staging System; *R-ISS*, Revised ISS. Staging systems were not pooled or converted. Cycle numbers describe the recorded assessment context, not necessarily completed cycles or uninterrupted treatment duration. Concomitant agents were explicitly mentioned in five records; absence of a mention does not establish monotherapy. Treatment continuation/discontinuation status was not consistently documented. Comorbidity categories are nonexclusive; absent questionnaire responses were not coded as absence of disease

**Fig. 1:**
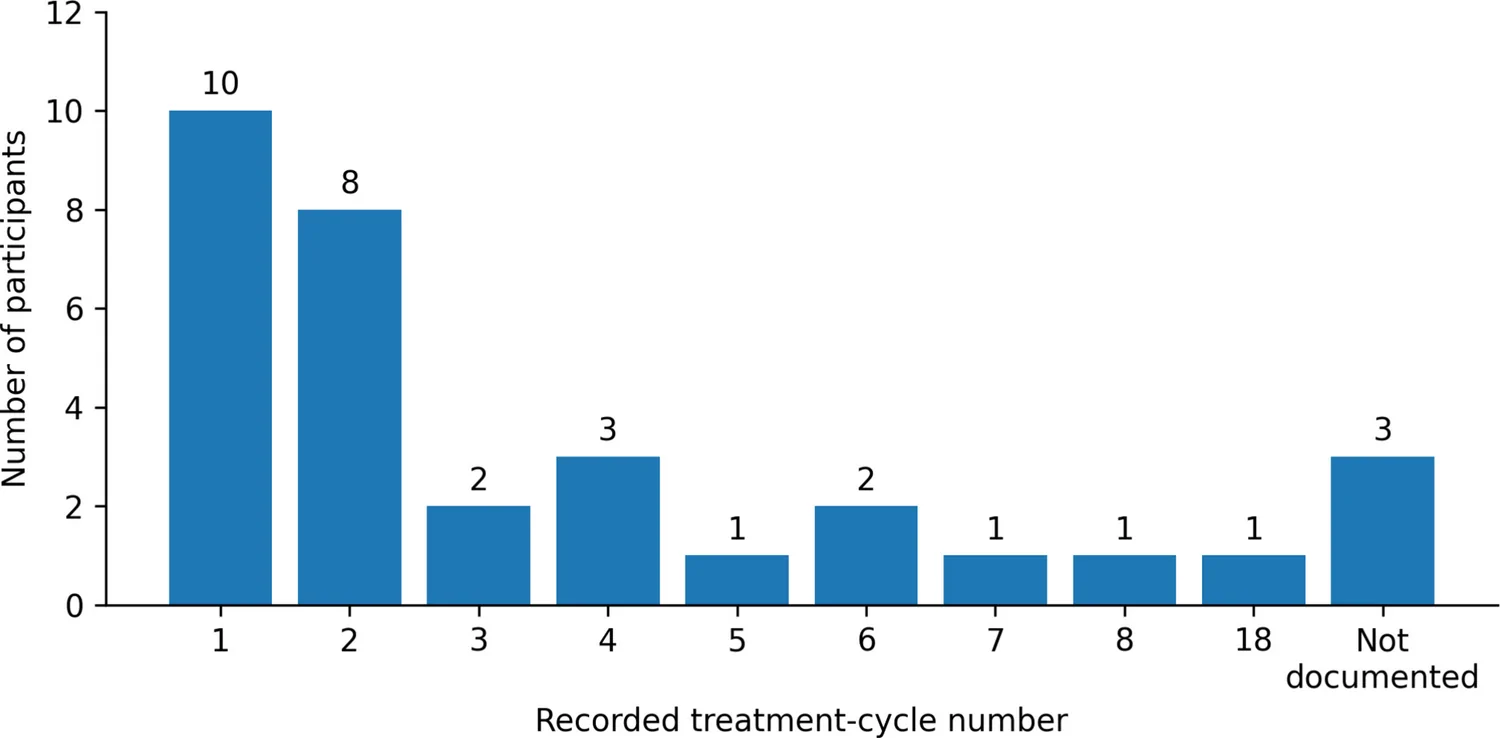
Recorded treatment-cycle numbers at assessment. Distribution of the cycle number documented in the treatment entry for all 32 participants. The “Not documented” category includes records without an interpretable cycle number. Bars show counts, not longitudinal observations or treatment-status groups. A recorded cycle number does not necessarily denote completed cycles or uninterrupted treatment exposure

An interpretable treatment-cycle number was documented for 29 participants: ten were recorded at cycle 1, eight at cycle 2, two at cycle 3, three at cycle 4, and six at later cycles. The median recorded cycle was 2 (IQR 1–4; range 1–18). Three records lacked an interpretable cycle number. These were heterogeneous assessment contexts, not serial observations of one cohort (Fig. 1).

### Taste performance and patient-reported burden

All 32 participants reported taste disturbance at some point during TAL therapy. The median recorded correct-identification count was 2.5 (IQR 0.75–7.25; observed range 0–14), with a mean of 4.47 (SD 4.49). Eight participants (25.0%) had no correct identifications. Nine (28.1%) reported a previous taste disturbance, while one history response was missing. Dry mouth was reported by 26 (81.3%), nail changes by 21 (65.6%), and dry or peeling skin by 15 (46.9%) (Table 2).

**Table 2:** Taste-identification performance, recalled course, nutritional changes, and treatment perceptions

Measure	Value
Taste assessment	
Taste disturbance reported during talquetamab therapy	32 (100.0%)
Recorded correct-identification count: median (IQR)	2.5 (0.75–7.25)
Recorded correct-identification count: mean (SD); range	4.47 (4.49); 0–14
No correct identifications recorded	8 (25.0%)
History of taste disturbance before this treatment	9 (28.1%)
Previous taste-disturbance history not recorded	1 (3.1%)
Recalled onset after first dose	
Within 14 days	23 (71.9%)
15 days to approximately one month	4 (12.5%)
Four to five weeks	1 (3.1%)
Other timing/pre-existing/not recorded	2/1/1
Improvement-related reports	
Explicit improvement mentioned in narrative item	8 (25.0%)
Fluctuating course without explicit improvement	1 (3.1%)
Affirmative improvement checklist response	2 (6.3%)
Response specifying a time of complete restoration	3 (9.4%)
Appetite and body weight	
Current appetite reduction	20 (62.5%)
Stable appetite	11 (34.4%)
Initial appetite reduction, subsequently resolved	1 (3.1%)
Directional weight loss since treatment initiation	16 (50.0%)
Magnitude among 16 reporting loss: median (IQR), kg	4.5 (2.0–8.5)
Weight gain/stable weight/nondirectional fluctuation	4/11/1
Other reported symptoms	
Dry mouth	26 (81.3%)
Nail changes	21 (65.6%)
Dry or peeling skin	15 (46.9%)
Unpleasant bitter taste	5 (15.6%)
Treatment perceptions	
Ever considered stopping talquetamab	10 (31.3%)
Taste explicitly cited among reasons for that consideration	7 (21.9% of cohort; 70.0% of 10)
Attributed taste changes at least partly to talquetamab	32 (100.0%)

Percentages use *n* = 32 unless stated otherwise. The aggregate correct-identification count is not a verified four-quality 0–16 score; no normative hypogeusia threshold was applied. Other onset timing refers to step-up dosing without an exact day and a few days after a full cycle without a first-dose interval. Improvement-related items are nonexclusive and sometimes discordant; none constitutes a longitudinally verified recovery rate. One weight-loss report described subsequent normalization; a 3–4-kg interval was summarized by its midpoint. Weight fluctuation without a directional loss was kept separate. Symptoms refer to reports at any time since treatment initiation, not necessarily current symptoms or clinician-graded adverse events. Treatment perceptions do not measure adherence or adjudicated discontinuation outcomes

Current impairment could be graded from explicit intensity descriptions in 27 participants. Twelve described severe and three very severe impairment; five described moderate, four mild, and three no impairment. Three responses did not specify a classifiable intensity, and two were missing. Eighteen participants (56.3%) reported changed food preferences or aversions. Savory/salty foods were mentioned as preferred by 13 (40.6%), and sweet foods, fruit, or fruit drinks by 12 (37.5%). Six (18.8%) mentioned aversion to sweet foods/drinks and four (12.5%) to alcoholic beverages (Table 3).

**Table 3:** Current taste-related impairment and food preferences

Patient-reported feature	*n* (% of 32)
Current impairment: coded intensity descriptions	
None	3 (9.4%)
Mild	4 (12.5%)
Moderate	5 (15.6%)
Severe	12 (37.5%)
Very severe	3 (9.4%)
Response did not specify a classifiable intensity	3 (9.4%)
No response recorded	2 (6.3%)
Food preferences and aversions	
Savory/salty foods mentioned as preferred	13 (40.6%)
Sweet foods, fruit, or fruit drinks mentioned as preferred	12 (37.5%)
Sweet foods/drinks mentioned as disliked	6 (18.8%)
Alcoholic beverages mentioned as disliked	4 (12.5%)
Reported a change in food preferences/aversions since treatment	18 (56.3%)

Impairment was elicited with an open question, not a validated five-point scale. Explicit intensity descriptions were coded 0–4 in 27 participants; nonspecific distress descriptions, “not that severe,” and missing answers were not assigned a grade. The coding rules and a sensitivity analysis are provided in Online Resource 1. Food categories are nonexclusive descriptive codes of current preferences; they do not independently establish a new preference, a change in nutrient intake, or the absence of unmentioned preferences

### Recalled onset and course

Twenty-three participants (71.9%) described onset within 14 days of the first TAL dose. Four (12.5%) reported 15 days to approximately 1 month and one (3.1%) 4 to 5 weeks. Other records described onset during step-up dosing without an exact day (*n* = 1), a few days after a full cycle without a first-dose interval (*n* = 1), pre-existing disturbance (*n* = 1), or no interpretable timing (*n* = 1).

Eight participants mentioned improvement in their narrative response and one described a fluctuating course without explicitly stating improvement. Only two endorsed improvement in the corresponding checklist item. Three supplied a time for complete restoration, but these responses did not establish sustained recovery or symptom absence at assessment. Narratives included changes between doses and after treatment cessation. Because timing, wording, and item responses were not concordant, no overall recovery rate or treatment-cessation effect was estimated.

### Appetite, weight, and treatment perceptions

Twenty participants (62.5%) reported current appetite reduction, 11 (34.4%) stable appetite, and one (3.1%) an initial reduction that had resolved. Sixteen (50.0%) reported directional weight loss since starting TAL, with a median loss of 4.5 kg (IQR 2.0–8.5; range 1–15); one of these described subsequent weight normalization. Four reported weight gain, 11 stable weight, and one nondirectional fluctuation. Intentionality and standardized serial weights were not available.

**Fig. 2:**
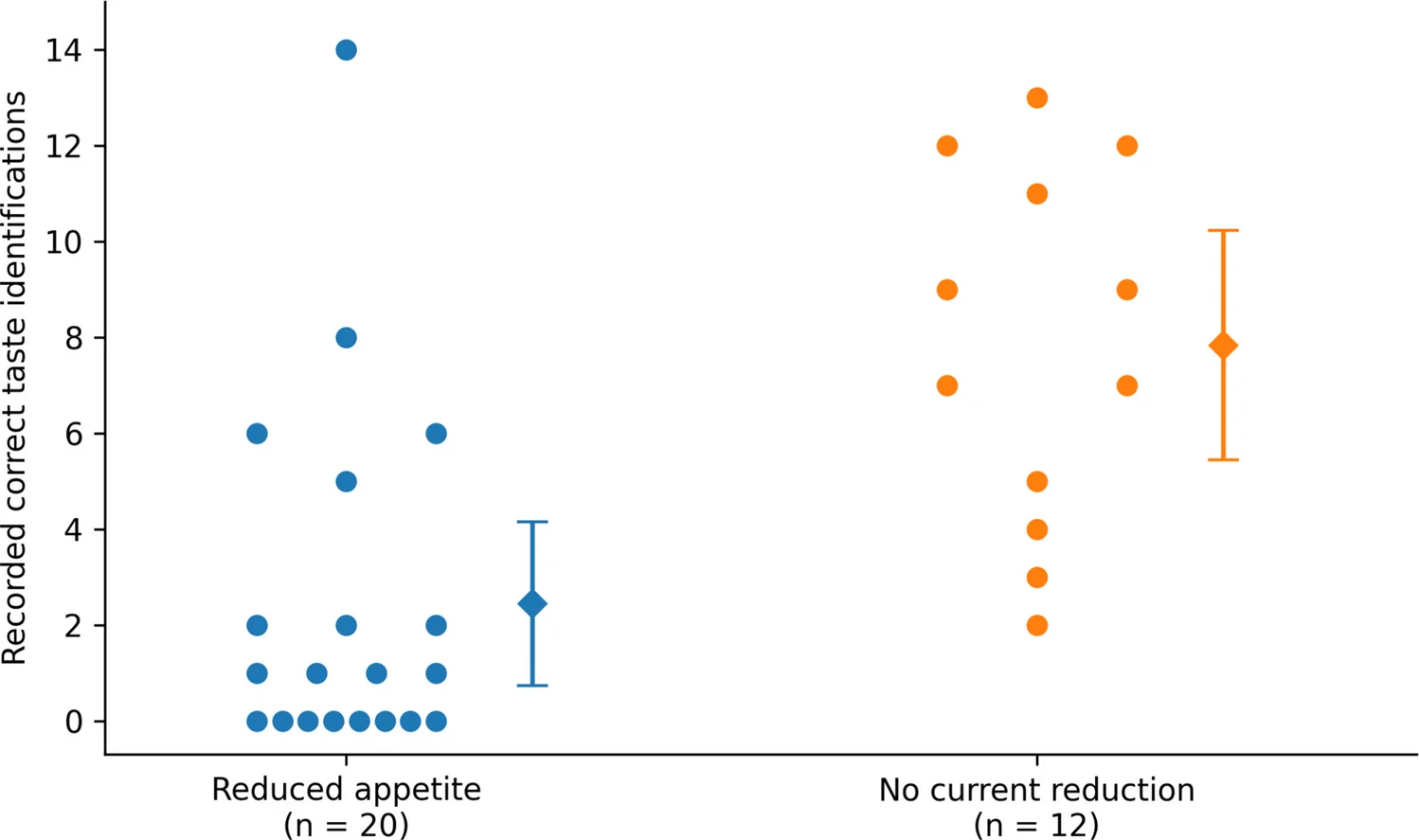
Taste-identification counts by current appetite status. Observed correct-identification counts in participants reporting reduced appetite (*n* = 20) or no current reduction (*n* = 12). The latter group includes one participant whose initial reduction had resolved. Circles represent individual observations; horizontal spreading separates identical counts without changing measured values. Diamonds with error bars indicate group means and 95% confidence intervals. The outcome is the recorded aggregate count, not a verified norm-referenced 0–16 score

Mean correct-identification counts were 2.45 (SD 3.65) with reduced appetite and 7.83 (SD 3.76) without current reduction: difference − 5.38 (95% CI − 8.20 to − 2.57; Welch *p* = 0.0006; Fig. 2). The point-biserial correlation was *r* =  − 0.589 (*p* = 0.0004). The association was also evident with a rank-based comparison (Mann–Whitney *p* = 0.0005) and after excluding the resolved appetite report (Welch *p* = 0.0015).

The binary appetite–weight-loss comparison was inconclusive (odds ratio 1.93; Fisher *p* = 0.473; *n* = 31). In contrast, participants reporting directional weight loss had higher EORTC appetite-loss scores (66.7 versus 33.3; difference 33.3 points, 95% CI 1.9–64.8; *p* = 0.039). The measures address different time frames and were not interchangeable. Ten participants (31.3%) had considered stopping TAL; seven explicitly cited taste disturbance among the reasons (21.9% of the cohort; 70.0% of those considering stopping). These reports did not establish actual nonadherence. All 32 attributed taste changes at least partly to TAL.

### Quality of life and exploratory associations

Mean EORTC global health status was 50.5 (SD 22.0). Role functioning had the lowest mean functional score (41.1), followed by social functioning (45.8); fatigue (53.5) and appetite loss (49.0) had the highest mean symptom scores. PHQ-4 indicated none/minimal distress in 17 participants (53.1%), mild distress in 10 (31.3%), moderate distress in five (15.6%), and severe distress in none (Table 4).

**Table 4:** EORTC QLQ-C30 scores and PHQ-4 distress categories

Outcome	Mean (SD) or *n* (%)
EORTC QLQ-C30: global health and functioning	
Global health status/QoL	50.5 (22.0)
Physical functioning	67.3 (22.1)
Role functioning	41.1 (33.3)
Emotional functioning	62.2 (28.7)
Cognitive functioning	68.2 (30.9)
Social functioning	45.8 (31.7)
EORTC QLQ-C30: symptoms and financial difficulties	
Fatigue	53.5 (27.9)
Nausea/vomiting	8.3 (15.3)
Pain	26.0 (36.4)
Dyspnea	21.9 (32.4)
Insomnia	22.9 (28.6)
Appetite loss	49.0 (45.6)
Constipation	11.5 (27.6)
Diarrhea	7.3 (16.4)
Financial difficulties	8.3 (20.7)
PHQ-4	
Total score (0–12)	2.44 (2.30)
None/minimal (0–2)	17 (53.1%)
Mild (3–5)	10 (31.3%)
Moderate (6–8)	5 (15.6%)
Severe (9–12)	0 (0.0%)

All scale summaries include 32 participants. EORTC QLQ-C30 scores range from 0 to 100; higher global health/functioning scores indicate better status, whereas higher symptom/financial-difficulty scores indicate greater burden. One out-of-range pain-item entry was treated as missing; for that participant, the pain scale used the other valid item under the EORTC half-item rule. Complete-pair sensitivity results are in Online Resource 1. PHQ-4 categories describe screening-score severity and are not psychiatric diagnoses. *SD*, sample standard deviation

Higher taste-identification counts correlated with better global health status (*r* = 0.406, 95% CI 0.066–0.661; *p* = 0.021; Online Resource 1, Figure S1). The Spearman result was similar (*rₛ* = 0.390; *p* = 0.027). Among 27 participants with classifiable current impairment, greater impairment correlated with lower taste counts (*rₛ* =  − 0.515; *p* = 0.006) and lower global health (*rₛ* =  − 0.566; *p* = 0.002). Women had higher mean counts than men (7.36 versus 2.95; difference 4.41, 95% CI 1.02–7.80; *p* = 0.014). These associations were unadjusted and exploratory. All other completed comparisons, including nonsignificant results, are reported in Online Resource 1.

## Discussion

This cross-sectional analysis characterizes patient-reported taste disturbance in a TAL-exposed clinical cohort and demonstrates associations of lower recorded taste-identification counts with reduced appetite and lower global health status. Its principal contribution is the within-cohort association between taste performance and appetite, supported by both mean-based and rank-based analyses, together with the distinction between symptom history, current impairment, and thoughts about stopping treatment. Universal symptom reporting in this selected sample should not be interpreted as the incidence of dysgeusia in all patients receiving TAL.

The findings are consistent with the clinical relevance of oral symptoms described during TAL treatment [1–3]. Laheij and van de Donk reported pre/post-treatment changes in taste performance and oral symptoms, while Naqvi and colleagues described weight loss and dysgeusia [2, 3]. Our earlier comparative report addressed differences between MM treatment groups [7]; it is cited as related work rather than treated as an independent validation dataset. The present analysis does not establish comparative toxicity, reproduce a pretreatment-to-treatment change, or provide a verified norm-referenced prevalence. Aggregate counts and normative four-quality scores should not be treated as interchangeable [8–10].

The appetite association persisted when the participant with resolved initial appetite loss was excluded. This supports an association with current appetite status rather than dependence on that single classification decision. Nevertheless, taste impairment and appetite reduction may share causes, including disease burden, other treatment effects, oral symptoms, medications, and comorbidity. Neither the cross-sectional association nor the patients’ attribution to TAL identifies the magnitude of a causal drug effect.

The distinction between appetite measures is informative. Directional weight loss was not clearly associated with the binary current appetite item, whereas the EORTC appetite-loss score was higher among participants reporting weight loss. These findings are not necessarily contradictory: a history of weight loss, current appetite classification, and a recent symptom-severity score capture different aspects and time windows. One participant reported weight normalization, and another reported only fluctuating weight. Without standardized baseline and follow-up weights, energy-intake data, or nutritional-risk assessment, neither malnutrition prevalence nor a taste-to-appetite-to-weight causal pathway can be inferred.

Taste-identification performance and current impairment were associated with global health, but these were unadjusted relationships within a small sample. Role and social functioning had the lowest mean functioning scores, while PHQ-4 scores showed heterogeneous distress rather than uniformly high psychological morbidity. A low PHQ-4 score does not rule out taste-specific frustration or loss of enjoyment. Likewise, considering treatment cessation is clinically relevant information but is not equivalent to a missed dose, nonadherence, or discontinuation caused by dysgeusia. The distinction matters when discussing supportive care with patients.

The early onset narratives justify attention to symptoms soon after treatment initiation, while the heterogeneity of food preferences argues against a uniform dietary prescription. Individualized exploration of tolerated flavors, textures, meal enjoyment, and hydration is consistent with the variation reported in chemotherapy populations [6, 14]. In practice, the combination of taste complaints, reduced intake, or weight change can prompt nutritional assessment and discussion of emotional burden. These are supportive-care considerations, not interventions shown effective by this study; neither dietary adjustments nor dose modification was tested prospectively here.

The available course descriptions also illustrate why a single visit cannot establish recovery. Improvement may have referred to one taste quality, temporary changes between doses, adaptation, or cessation of therapy. Discordance between checklist answers and narratives prevents a reproducible single recovery endpoint. Future studies should distinguish partial improvement, temporary normalization, and sustained recovery, specify the reference date, and obtain serial taste and patient-reported measurements before, during, and after treatment.

### Limitations

The sample was small and from one center, with no pretreatment taste assessment or contemporaneous comparator. The number approached or eligible but not enrolled was not documented in the analytic records, so selection and survivorship bias cannot be quantified. Patients were assessed at heterogeneous treatment stages, and current treatment status could not be classified consistently. Different staging systems and incompletely documented concomitant therapy limited clinical adjustment. Findings are therefore not population-level incidence estimates or independent evidence of treatment causality.

Several measurement limitations affect interpretation. Taste-quality-specific responses were unavailable, precluding verification of a four-quality score, normative classification, and modality-specific comparisons. Current impairment and food preferences required post hoc coding of a nonvalidated questionnaire; five impairment responses were missing or not classifiable, and coding uncertainty was examined in sensitivity analysis. Recalled onset, weight change, and improvement are vulnerable to recall and interpretation differences. One invalid EORTC pain-item entry required the standard partial-scale rule, and pain-related results were sensitive to complete-pair restriction. Olfaction, salivary function, detailed dietary intake, and body composition were not systematically measured.

Multiple associations were examined without multiplicity correction, and the small cohort did not support reliable confounder adjustment. Confidence intervals quantify sampling uncertainty under the statistical model but do not resolve measurement error or residual confounding. Sex differences, QoL correlations, and other secondary results should consequently be treated as hypothesis-generating. Longitudinal studies with predefined coding, preserved item-level taste responses, standardized nutritional measurements, and clearly documented treatment exposure are needed to evaluate recovery and supportive-care strategies.

## Conclusion

Among these 32 TAL-exposed patients, taste complaints were common, and lower recorded taste-identification counts were associated with current appetite reduction and lower global health status. Half reported directional weight loss, and seven explicitly linked taste disturbance to consideration of stopping treatment. These findings support clinical attention to taste-related burden, while the cross-sectional design and limitations of the retained records preclude norm-based hypogeusia estimates, causal attribution, sustained-recovery rates, or claims of improved adherence through supportive care.

## Supplementary Information

Below is the link to the electronic supplementary material.

ESM 1 (DOCX 131 KB)

## Data Availability

The data supporting the findings of this study are available from the corresponding author upon reasonable request.
