# Supplementary material for: Gustatory dysfunction under talquetamab therapy in multiple myeloma: a cross-sectional study of patient-reported course, nutritional changes, and quality of life
Source: Support Care Cancer. 2026 Oct 5;34(10):1059. doi: 10.1007/s00520-026-11288-4 (PMC13638732; doi:10.1007/s00520-026-11288-4)
Supplement: Supplementary file 1 [file 520_2026_11288_MOESM1_ESM.docx]

**Online Resource 1**

**Supplementary methods and complete exploratory analyses**

This supplement documents coding, complete analyses, data limitations, and sensitivity checks for all 32 participants. Smaller analysis-specific denominators reflect missing or nonclassifiable variables, not exclusion from the cohort. No identifying participant information is included.

**Table S1. Variable definitions and data-handling rules**

| **Variable or issue** | **Rule applied** |
| --- | --- |
| Analysis set | Two paired records per participant: taste/clinical questionnaire and PHQ-4/EORTC items. All 32 matched pairs were retained. Repeated formatting and isolated numbers below the patient block were not additional participants. |
| Taste performance | Use the stored aggregate correct-identification count without subtracting an assumed umami contribution. Observed range 0–14; total across participants 143. Per-quality/per-concentration records are unavailable. The conventional 0–16 total, hypogeusia prevalence, and quality-specific rates cannot be reconstructed. |
| Recorded cycle | Extract an explicitly recorded cycle number. Do not infer cycle 1 from a day-only entry, a cessation entry without a cycle, or re-step-up treatment. There are 29 interpretable values and three unknowns. Cycle number is not duration of treatment. |
| Treatment status | Four regimen entries explicitly mention cessation; two additional questionnaire narratives mention it. The timing is not uniformly clear, and some entries conflict. No common current-treatment-status variable was constructed; no cessation effect or active-treatment-only subgroup is reported. |
| Staging | Use unqualified I, II, or III entries under the ISS header as ISS (n = 23). Keep explicitly labeled R-ISS (n = 2) and Durie–Salmon (n = 5) separate. A qualitative FISH-risk statement and a missing entry are not stage values. The ISS comparison uses 12 stage-I versus 11 stage-II/III records only. |
| Appetite | Reduced = a current reduction reported in the appetite-change item (20). Comparison = stable appetite (11) plus initial reduction followed by normalization (1). The latter participant is excluded in sensitivity analysis. The EORTC appetite item is a separate recent symptom-severity measure. |
| Weight change | Directional loss since treatment initiation: 16, including one report of later normalization. Gain: 4; stable: 11; nondirectional fluctuation: 1. The fluctuation report is not automatically assigned to loss. Binary weight-loss comparisons use 31 classifiable records. Reported intentionality is not available. |
| Weight-loss magnitude | Use the stated magnitudes in the 16 directional-loss reports. Represent the sole 3–4-kg interval by 3.5 kg for summaries. This does not change the reported median or IQR. No baseline body weight or relative percentage loss is reconstructed from current weight. |
| Onset categories | Within 14 days: 23, including immediate onset, hours, a few days, and intervals ending within 14 days. Fifteen days to approximately one month: 4 (20 days, 3 weeks, 4 weeks, 1 month). Four to five weeks: 1. Step-up without an exact interval: 1; a few days after a full cycle: 1; pre-existing: 1; not recorded: 1. No exact onset median was estimated. |
| Improvement and restoration | Eight narrative responses explicitly mention some improvement; one describes a fluctuating course. Only two affirm the improvement checklist. Three provide a time in the complete-restoration item. Responses can overlap and conflict. These are reports from distinct questions, not interchangeable event indicators; no uniform recovery endpoint, time-to-recovery analysis, or recovery-group hypothesis test is constructed. |
| Current impairment: 0–4 coding | The source question is open-ended. None (0): explicit no impairment. Mild (1): hardly/little/a little. Moderate (2): moderate/medium. Severe (3): strong, quite strong, substantial, severe, or very impaired without an explicit very-severe category. Very severe (4): explicitly very strong impairment. Counts: 3, 4, 5, 12, and 3, respectively; n = 27. |
| Unclassifiable current impairment | Annoyed, very stressed, and not that severe do not specify an unambiguous grade and remain ungraded (n = 3). Two absent answers remain missing. They are not coded as zero. A sensitivity analysis codes only not that severe as mild, giving n = 28; the other four remain ungraded. |
| Savory/salty preference code | Descriptive category includes explicit savory/salty/hearty preferences and named meat, eggs, cheese snacks, soup, noodles, potatoes, or mashed potatoes. Vegetables alone and unspecified soft pastry are not automatically assigned. Thirteen participants mention at least one qualifying food; this is not a quantitative nutrient-intake measure. |
| Sweet preference and aversion codes | Preferred sweets, dessert, jam, fruit, or fruit drinks are grouped descriptively (12); fruit explicitly described as sour is not counted as sweet preference. Disliked sweets/fruit drinks/cocoa are grouped descriptively (6). Alcohol aversion is separate (4). Generic dislike of everything is not recoded as a specific category. Categories are nonexclusive and do not establish new preferences. |
| Any preference change | Use the explicit yes/no answer to the preference-change item: 18 yes. Do not infer a changed preference solely from current food lists. |
| Symptoms | Use the dedicated item asking about symptoms noticed since TAL initiation: taste change 32, dry mouth 26, nail changes 21, dry/peeling skin 15, and unpleasant bitter taste 5. Do not add a symptom solely because it is mentioned in another item or infer current absence from a past-symptom list. |
| Stopping treatment | Use the actual consideration item (10 yes) and its immediately following reasons item. Taste disturbance is explicit in seven of those ten reason responses. Do not use a hypothetical future-trigger item or general poorer QoL to assign taste as an actual reason. These are treatment perceptions, not adjudicated adherence or discontinuation events. |
| Comorbidities | Recognize explicit terms and abbreviations: polyneuropathy/PNP, diabetes/DM, reflux, stroke, COVID-19. Three absent responses are missing, not negative. Descriptive counts use the cohort denominator; reflux and comorbidity-count analyses restrict to the 29 completed responses. The non-COVID count sums the other four listed conditions. |
| Age and dates | Calculate completed years from the birth and assessment dates in the clinical sheet. The second sheet contains two missing birth dates, but the clinical sheet contains both. One assessment date is chronologically unusual; it is retained as recorded, not silently moved to another year. The main text reports the documented assessment years, 2024–2025. |
| EORTC QLQ-C30 | The file explicitly stores questions 1–28 as 0–3. Standardized function scores = 100 × (1 − mean/3); symptom scores = 100 × mean/3. Global health = 100 × (mean of questions 29–30 − 1)/6. Use the version-3.0 item sets in the EORTC manual; sample SD uses n−1. The invalid value 11 in one pain item is missing, not guessed to be 1. The other pain item supplies that participant’s scale under the half-item rule. |
| PHQ-4 | Sum the four valid 0–3 responses, yielding a 0–12 score. Categories: 0–2 none/minimal, 3–5 mild, 6–8 moderate, 9–12 severe. All 32 records are complete; these categories do not establish a psychiatric diagnosis. |
| Analysis scope | All coding decisions were made during revision from the retained records and are post hoc. They are not a preregistered coding manual or a validated questionnaire. No assessor-agreement statistic or blinded dual coding is claimed. Tests are two-sided and exploratory; no multiplicity-adjusted or causal inference is made. |

EORTC scoring follows the version-3.0 algorithm and missing-item rule in the EORTC QLQ-C30 Scoring Manual (Fayers et al., 2001; full citation in the main manuscript). The original clinical workbook was not overwritten.

**EORTC QLQ-C30 item sets**

Physical functioning: 1–5; role: 6, 7; emotional: 21–24; cognitive: 20, 25; social: 26, 27. Fatigue: 10, 12, 18; nausea/vomiting: 14, 15; pain: 9, 19; dyspnea: 8; insomnia: 11; appetite loss: 13; constipation: 16; diarrhea: 17; financial difficulties: 28. Global health/QoL: 29, 30. Item labels were checked against the source workbook.

**Table S2. All completed exploratory association analyses**

| **Association / contrast** | **Test** | **n** | **Effect estimate [95% CI]** | **p** |
| --- | --- | --- | --- | --- |
| S2.1. Taste count: women − men | Welch | 11/21 | Means 7.36 (4.61) vs 2.95 (3.69); Δ 4.41 [1.02, 7.80] | 0.014 |
| S2.2. Age × taste count | Pearson | 32 | r = −0.182 [−0.499, 0.178] | 0.320 |
| S2.3. Current impairment grade × taste count | Spearman | 27 | rₛ = −0.515 | 0.006 |
| S2.4. Any consideration of stopping × female sex | Fisher exact | 32 | OR 0.36; cells [2, 8; 9, 13] | 0.425 |
| S2.5. Directional weight loss × reduced appetite | Fisher exact | 31 | OR 1.93; cells [11, 5; 8, 7] | 0.473 |
| S2.6. EORTC appetite loss: weight loss − no loss | Welch | 16/15 | Means 66.67 (43.89) vs 33.33 (41.79); Δ 33.33 [1.86, 64.80] | 0.039 |
| S2.7. Taste count: reduced appetite − no current reduction | Welch | 20/12 | Means 2.45 (3.65) vs 7.83 (3.76); Δ −5.38 [−8.20, −2.57] | 0.0006 |
| S2.8. Reduced appetite (1) × taste count | Pearson | 32 | r = −0.589 [−0.778, −0.303] | 0.0004 |
| S2.9. Taste count: ISS I − ISS II/III | Welch | 12/11 | Means 4.50 (4.42) vs 5.55 (5.07); Δ −1.05 [−5.20, 3.11] | 0.605 |
| S2.10. Global health: partnership/marriage − other status | Welch | 26/6 | Means 48.72 (23.65) vs 58.33 (10.54); Δ −9.62 [−22.89, 3.66] | 0.146 |
| S2.11. EORTC appetite loss × nausea/vomiting | Pearson | 32 | r = 0.039 [−0.314, 0.382] | 0.834 |
| S2.12. EORTC diarrhea: weight loss − no loss | Welch | 16/15 | Means 6.25 (18.13) vs 8.89 (15.26); Δ −2.64 [−14.93, 9.65] | 0.664 |
| S2.13. Taste count × global health | Pearson | 32 | r = 0.406 [0.066, 0.661] | 0.021 |
| S2.14. Taste count: reflux recorded − reflux not recorded | Welch | 6/23 | Means 6.33 (5.89) vs 4.39 (4.27); Δ 1.94 [−4.23, 8.11] | 0.475 |
| S2.15. Number of recorded non-COVID comorbidities × global health | Pearson | 29 | r = −0.094 [−0.445, 0.283] | 0.629 |
| S2.16. EORTC social functioning × global health | Pearson | 32 | r = 0.473 [0.148, 0.705] | 0.006 |
| S2.17. EORTC physical functioning × global health | Pearson | 32 | r = 0.578 [0.287, 0.771] | 0.0005 |
| S2.18. EORTC fatigue × global health | Pearson | 32 | r = −0.252 [−0.552, 0.106] | 0.165 |
| S2.19. EORTC appetite loss × global health | Pearson | 32 | r = −0.339 [−0.615, 0.011] | 0.058 |
| S2.20. EORTC pain × global health | Pearson | 32 | r = −0.370 [−0.637, −0.025] | 0.037 |
| S2.21. Current impairment grade × global health | Spearman | 27 | rₛ = −0.566 | 0.002 |

All p values are two-sided, nominal, and unadjusted for multiplicity. Welch contrasts are first-listed group minus second-listed group; group summaries are mean (sample SD), and n shows the two group sizes. Pearson intervals use Fisher’s z transformation. Spearman p values are asymptotic; no confidence interval is asserted for these ordinal associations. In 2×2 tables, rows represent first variable yes/no and columns second variable yes/no; OR is the sample odds ratio. Current impairment is the post hoc 0–4 grade in 27 classifiable responses. Absence of a recorded comorbidity is not equated with clinically verified absence. No p value from the original submission was retained without recalculation.

**Table S3. Analyses not reconstructible from the retained records**

| **Analysis or claim** | **Reason not reported as a result** |
| --- | --- |
| Norm-referenced hypogeusia, normal-versus-impaired comparisons with QoL or PHQ-4, and taste-quality-specific performance | The retained aggregate count does not verify a four-quality total, and responses by quality/concentration are absent. Neither a 0–16 total nor its diagnostic threshold was reconstructed. |
| Sex, age, education, or global-health comparisons using a binary improvement group | The improvement checklist and course narratives are not concordant and do not identify a uniform current or sustained-recovery endpoint. |
| Treatment-cessation associations with improvement or taste count | Current ongoing/discontinued status is not consistently recorded, and retrospective cessation references have heterogeneous timing. |
| Taste-count comparison by dysgeusia-related stopping consideration restricted to ongoing treatment | The required current-treatment-status restriction cannot be applied reliably. The seven explicit reasons are retained as a descriptive cohort finding. |
| Longitudinal recovery, nutritional causality, relative baseline weight loss, and observed nonadherence | There are no repeated standardized taste/weight measurements, no verifiable baseline-weight series, and no adjudicated adherence outcome in these records. Narrative reports are not substituted for these endpoints. |

These restrictions are based on variable availability and interpretability, not on the direction or statistical significance of a result. Descriptive reports from the relevant questionnaire items remain in the main text and Table 2.

**Table S4. Sensitivity analyses**

| **Analysis** | **n** | **Result** |
| --- | --- | --- |
| Taste counts by appetite: Mann–Whitney U | 20 / 12 | U = 31.0; two-sided p = 0.0005 (tie-corrected asymptotic test) |
| Taste counts by appetite: exclude resolved initial appetite reduction | 20 / 11 | Mean difference −4.91; 95% CI −7.71 to −2.11; Welch p = 0.0015 |
| Taste counts and global health: rank correlation | 32 | Spearman rₛ = 0.390; p = 0.027 |
| Grade “not that severe” as mild: impairment and taste count | 28 | Spearman rₛ = −0.434; p = 0.021 (main n = 27: −0.515; p = 0.006) |
| Same alternative grade: impairment and global health | 28 | Spearman rₛ = −0.559; p = 0.002 (main n = 27: −0.566; p = 0.002) |
| Weight fluctuation assigned to loss | 32 | Weight-loss/appetite OR 2.10; Fisher p = 0.467 |
| Weight fluctuation assigned to no loss | 32 | Weight-loss/appetite OR 1.71; Fisher p = 0.716 |
| Exclude resolved weight-loss report and nondirectional fluctuation | 30 | Weight-loss/appetite OR 2.41; Fisher p = 0.450 |
| EORTC appetite severity: exclude resolved weight-loss report | 15 / 15 | Mean difference 37.78; 95% CI 6.62 to 68.94; Welch p = 0.019 |
| Pain scale: complete two-item records only | 31 | Mean 26.88 (SD 36.69), versus 26.04 (36.40) with the half-item rule in n = 32 |
| Pain and global health: complete two-item records only | 31 | Pearson r = −0.350; 95% CI −0.627 to 0.005; p = 0.054 (main p = 0.037) |

These sensitivity checks assess dependence on coding or model assumptions; they do not remove confounding or validate an uncertain variable. The pain-global-health association changes from nominal p = 0.037 to p = 0.054 when the incomplete pain pair is excluded, illustrating its fragility.

**Figure S1. Taste-identification counts and global health status**

Paired observed values from all 32 participants. Marker area is proportional to the number of participants sharing an identical pair of values. The horizontal axis is the recorded aggregate correct-identification count; the vertical axis is EORTC QLQ-C30 global health status/quality of life (0–100).

For Figure S1, Pearson r = 0.406 (95% CI 0.066–0.661; p = 0.021; n = 32); Spearman rₛ = 0.390 (p = 0.027). These are unadjusted associations, not estimates of a causal effect.
